# Structural similarities and functional differences clarify evolutionary relationships between tRNA healing enzymes and the myelin enzyme CNPase

**DOI:** 10.1186/s12858-017-0084-2

**Published:** 2017-05-16

**Authors:** Gopinath Muruganandam, Arne Raasakka, Matti Myllykoski, Inari Kursula, Petri Kursula

**Affiliations:** 1 Centre for Structural Systems Biology - Helmholtz Centre for Infection Research, German Electron Synchrotron (DESY), Hamburg, Germany; 2 Faculty of Biochemistry and Molecular Medicine & Biocenter Oulu, University of Oulu, Oulu, Finland; 3 Department of Biomedicine, University of Bergen, Bergen, Norway

**Keywords:** Cyclic phosphodiesterase, Polynucleotide kinase, Substrate specificity, Protein structure, 2H family, Evolution, tRNA splicing

## Abstract

**Background:**

Eukaryotic tRNA splicing is an essential process in the transformation of a primary tRNA transcript into a mature functional tRNA molecule. 5′-phosphate ligation involves two steps: a healing reaction catalyzed by polynucleotide kinase (PNK) in association with cyclic phosphodiesterase (CPDase), and a sealing reaction catalyzed by an RNA ligase. The enzymes that catalyze tRNA healing in yeast and higher eukaryotes are homologous to the members of the 2*H* phosphoesterase superfamily, in particular to the vertebrate myelin enzyme 2′,3′-cyclic nucleotide 3′-phosphodiesterase (CNPase).

**Results:**

We employed different biophysical and biochemical methods to elucidate the overall structural and functional features of the tRNA healing enzymes yeast Trl1 PNK/CPDase and lancelet PNK/CPDase and compared them with vertebrate CNPase. The yeast and the lancelet enzymes have cyclic phosphodiesterase and polynucleotide kinase activity, while vertebrate CNPase lacks PNK activity. In addition, we also show that the healing enzymes are structurally similar to the vertebrate CNPase by applying synchrotron radiation circular dichroism spectroscopy and small-angle X-ray scattering.

**Conclusions:**

We provide a structural analysis of the tRNA healing enzyme PNK and CPDase domains together. Our results support evolution of vertebrate CNPase from tRNA healing enzymes with a loss of function at its N-terminal PNK-like domain.

**Electronic supplementary material:**

The online version of this article (doi:10.1186/s12858-017-0084-2) contains supplementary material, which is available to authorized users.

## Background

Eukaryotic tRNA processing is an essential process, by which newly synthesized immature pre-tRNA matures into functional tRNA. The processing of tRNA begins with endonucleolytic cleavage of the pre-tRNA into an intron and two tRNA halves that are healed and sealed by tRNA splicing. The 5′-phosphate ligation pathway, used by yeast and plants, involves the 5′-phosphate of the 3′-tRNA half as the junction phosphate of the new phosphodiester linkage [1, 2]. 5′-phosphate ligation requires three enzymatic activities: a cyclic phosphodiesterase (CPDase), a polynucleotide kinase (PNK), and a ligase [1, 3–5]. In contrast to yeast and plants, animal cells employ two different ligation pathways. The 3′-phosphate ligation pathway utilizes the 3′-phosphate of the 5′-tRNA half as the junction phosphate; this pathway was first detected in HeLa cell extracts [6]. The second pathway used by animal cells is a yeast-type 5′-phosphate ligation pathway, which has been detected in HeLa cell extracts and in the lancelet, *Branchiostoma floridae* [7, 8]. tRNA healing enzymes appear to be distant homologues of the 2*H* phosphoesterase superfamily, which is defined by the presence of two conserved H-x-T/S-x (x is a hydrophobic residue) motifs [8–10].

The central kinase module of yeast (*Saccharomyces cerevisiae*) Trl1 and the N-terminal PNK domain of lancelet PNK/CPDase show resemblance to the bacteriophage T4 PNK and belong to the P-loop phosphotransferase superfamily. They contain the signature Walker A motif (P-loop), which is an NTP-binding site in most NTP-dependent phosphotransferases [8, 11–15]. Initial functional analysis of the kinase module of yeast Trl1, using single alanine mutations in the P-loop, revealed that the P-loop motif (^401^GCGKT^405^) is a determinant of NTP binding [16]. GTP is the preferred *in vivo* physiological substrate, and yeast Trl1 contains a single NTP binding site [16]. GTP dependence of the yeast Trl1 kinase domain has also been verified *in vitro* [17].

The CPDase domain of both yeast Trl1 and lancelet PNK/CPDase bears resemblance to phosphoesterases of the 2*H* superfamily [8–10]. Although 5′-P RNA ligases of fungi, plants, and animals share essential mechanistic features and key residues required for their PNK and CPDase activities, their overall sequence similarity is low. The presence of the P-loop or Walker A motif (G-x-G-K-T/S, x is any residue) in the N-terminal domain and the two H-x-T/S-x motifs in the C-terminal domain of vertebrate 2′,3′-cyclic nucleotide 3′-phosphodiesterase (CNPase) indicates that the PNK/CPDase domains of yeast Trl1 (*Sc* PNK/CPDase) and the lancelet (*Bf*) PNK/CPDase are homologues of vertebrate CNPase [8, 18].

In most vertebrates, CNPase is abundantly expressed in the myelin sheath, a multilayered proteolipid membrane system. CNPase constitutes 4% of total myelin protein in the central nervous system (CNS) and 0.4% in the peripheral nervous system (PNS) [19]. CNPase-deficient mice develop progressive motor deficits and die prematurely due to diffuse brain axonal swelling and neurodegeneration [20]. However, it is not known whether the late onset of neurodegeneration is caused by the absence of the CNPase protein as a structural component of the myelin sheath or the absence of CNPase enzymatic activity. CNPase has been implicated in neurodegenerative disorders, such as multiple sclerosis and schizophrenia [21, 22]. A recent study showed that CNPase inhibits the assembly of infectious particles of several primate lentiviruses, including the human immunodeficiency viruses HIV-1 and HIV-2, by binding to the structural protein Gag [23].

The enzymatic activity of CNPase, the hydrolysis of 2′,3′-cyclic nucleotides to 2′-nucleotides, was detected in brain tissue in the 1960s [24]. Atomic structures of the C-terminal catalytic domain of human, rat, and mouse CNPase have illuminated the catalytic role of the H-x-(T/S)-x motifs and other functionally important groups in the active site [18, 25–29]. The structure and function of the N-terminal domain of CNPase remain poorly characterized. The expression and purification of the N-terminal domain is difficult compared to the C-terminal domain, limiting the availability and characterization of the molecule [30]. Although the N-terminal domain of CNPase contains the NTP-binding P-loop motif, and a study by Stingo *et al*. claims that nucleotide binding occurs in the N-terminal domain, it has not been experimentally proven, whether the interaction and hydrolysis take place exclusively in the N-terminal domain, as only full-length CNPase was used in the experiments [31]. The N-terminal domain of CNPase binds RNA, mediates dimerization, and interacts with calmodulin (CaM) in a calcium-dependent manner [27, 32]. CNPase has recently been found to bind microfilaments and act as an antagonist for myelin basic protein in myelin membrane compaction [33], suggesting a structural role – possibly independent of enzymatic CNPase activity.

Although the structure of the C-terminal phosphodiesterase domain of vertebrate CNPase has been determined, no high-resolution structures of either full-length CNPase or its N-terminal domain are available. The functional properties of vertebrate CNPase, such as interactions with membranes, cytoskeletal proteins, and RNA have been studied; however, the link between its function in the myelin sheath and its role in RNA binding is not clear. Hence, structural and functional characterization of PNK/CPDase would benefit, not only the field of tRNA splicing, but also studies on other members of the 2*H* phosphoesterase family. Since *Bf*PNK/CPDase could be involved in a new class of 5′-P RNA ligation, characterization of this enzyme might improve our understanding of the growing complexity of animal tRNA splicing. Despite genetic and biochemical analysis of PNK and CPDase domains of yeast Trl1, no structural information is available for any domain of the enzyme. Therefore, detailed biophysical and biochemical analysis of *Sc*Trl1 PNK/CPDase and *Bf*PNK/CPDase is warranted. We generated multiple expression constructs encoding different segments of the PNK/CPDase domains of yeast Trl1, as well as full-length lancelet PNK/CPDase. An array of biophysical and biochemical methods was employed to characterize the proteins, including assays for activity, thermal stability, and folding of the PNK/CPDase domains.

## Methods

### Sequence and ligation independent cloning

The Protein Crystallographic Construct Design (ProteinCCD) metaserver was used to choose fragments from different domains of yeast and lancelet healing enzymes based on information acquired from various prediction servers for secondary structure, disorder, coiled coils, transmembrane segments, conserved domains, and domain linkers [34]. Multiple expression constructs encoding the PNK and CPDase domains of yeast tRNA ligase and full-length lancelet PNK/CPDase were assembled by sequence and ligation independent cloning (SLIC). The primers used are listed in (Additional file 1). Briefly, the DNA fragments of interest were amplified by polymerase chain reaction (PCR) from pET28a and pET20b plasmids harbouring *Sc*Trl1 and *Bf*PNK/CPDase, respectively [8]. The amplified products and linearized pET-his3C-LIC-amp vector (a kind gift from the Netherlands Cancer Institute) were separately treated with T4 DNA polymerase. The insert and vector were annealed, and the resulting plasmid was used to transform *E. coli* NEB5α cells (New England Biolabs, Germany). Transformed colonies were screened by colony PCR for recombinant plasmids that were purified, verified by sequencing, and used for protein expression.

### Protein expression and purification

Large-scale expression of *Sc*Trl1 PNK/CPDase, *Sc*Trl1 CPDase, and *Bf*PNK/CPDase was performed in *E. coli* Rosetta(DE3) cells (Novagen, Germany) cultured in LB medium containing 100 μg/ml ampicillin and 34 μg/ml chloramphenicol. After reaching an OD_600_ of 0.5–0.6, expression was induced with 0.15 mM isopropyl β-D-1 thiogalactopyranoside (IPTG) for 16 h at +18 °C. The cells were harvested by centrifugation and resuspended in lysis buffer (50 mM HEPES, pH 7.5, 200 mM NaCl, 10 mM β-mercaptoethanol (β-ME), 20 μg/ml DNase, Ambion RNase cocktail containing RNase A and RNase T1(Life Technologies, Germany), 5 mM MgCl_2_, and cOmplete mini EDTA-free protease inhibitors (Roche, Germany)). The suspension was incubated for 20 min at +4 °C on a tube rotator. The cells were disrupted by sonication, and debris was removed by centrifugation at 35,000 *g* for 30 min at +4 °C. The supernatant was applied to a gravity-flow Ni-NTA column, pre-equilibrated with lysis buffer. The columns were rotated horizontally for 1 h to ensure binding of the protein to the matrix. The column was washed with lysis buffer containing 50 mM imidazole, and bound protein was eluted with lysis buffer including 500 mM imidazole. The eluted fractions were studied by SDS-PAGE, and the fractions containing the protein of expected size were pooled and dialyzed against the lysis buffer (without imidazole). The N-terminal hexahistidine tag was cleaved using recombinant 3C protease at +4 °C overnight. The cleaved protein was further purified by Ni-NTA affinity chromatography and dialyzed against the lysis buffer without imidazole. The dialyzed proteins were concentrated and applied either to a HiLoad 16/60 Superdex 200 preparative grade column (column volume: 120 ml; injection volume: 1 ml) or to a Superdex 75 10/300 GL analytical grade column (column volume: 24 ml; injection volume: 100 μl), pre-equilibrated with 50 mM HEPES (pH 7.5), 200 mM NaCl, 10 mM β-ME. Equilibration, injection, and elution were all carried out at a flow rate of 1 ml/min. The protein-containing peaks were analyzed by SDS-PAGE, and the fractions containing the proteins of interest were pooled, concentrated, flash-frozen, and stored at −80 °C. The identity of the purified proteins was verified by tryptic peptide mapping using mass spectrometry at the Biocenter Oulu Proteomics Core Facility.

Full-length mouse CNPase (*Mm*CNPase, residues 20–420 of isoform 2), the N-terminal PNK domain (*Mm*CNP_N, residues 20–180), and the C-terminal catalytic CPDase domain (*Mm*CNP_C, residues 179–398) were purified using Ni-NTA chromatography, followed by His-tag cleavage using TEV protease, a second Ni-NTA step, and size exclusion chromatography (SEC), essentially as described before [30].

### Synchrotron radiation circular dichroism spectroscopy

The folding of *Sc*Trl1 PNK/CPDase, *Bf*PNK/CPDase, and *Sc*Trl1 CPDase was studied by SRCD spectroscopy on the UV-CD12 beamline at the ANKA Synchrotron. The proteins purified by SEC were dialyzed into a buffer compatible with SRCD analysis (50 mM potassium phosphate, pH 6.5). The range of protein concentrations used was between 3.5 and 5 mg/ml. Three spectra were collected at a scan rate of 14 nm/min, in a demountable 13-μm CaF_2_ cuvette at a wavelength range between 260 and 170 nm at 1 nm intervals. A spectrum from the corresponding buffer was measured for all the samples and used for background correction. The data were processed with CDtool [35] and analyzed using Dichroweb [36].

### PNK activity assay

The PNK activity of the N-terminal PNK domain of *Sc*Trl1 PNK/CPDase and *Bf*PNK/CPDase was assayed using the synthetic oligonucleotides ribo A_20_ (A_20_) and deoxy A_20_ (dA_20_), and a mixture of the two (A_20_ + dA_20_). T4 PNK and *Sc*Trl1 CPDase were used as positive and negative controls, respectively. The reaction mixture contained 70 mM Tris, pH 7.5, 10 mM MgCl_2_, and 5 mM DTT, 2 μg of enzyme, and 100 pmol A_20_ or dA_20_ or both. 55 pmol of radioactively labelled [γ^32^P] ATP were added to each reaction mixture, and the volume of the mixtures was adjusted to 40 μl with diethylpyrocarbonate-treated water. The samples were incubated at +37 °C, and 8-μl aliquots were removed at different time points (1, 15, 30, and 60 min). Each sample was quenched with 8 μl of 2X urea sample loading buffer (Invitrogen Novex) and heated at +90 °C for 4 min. The samples were analyzed by electrophoresis using a 15% Mini-PROTEAN TBE-Urea gel (Bio-Rad), at 150 V for 85 min, and visualized using PhosphorImager analysis.

### CPDase activity assay

Kinetic measurements were carried out as previously described [30, 37]. In this assay, CPDase hydrolyzes the phosphodiester bond in β-nicotinamide adenine dinucleotide 2′,3′-cyclic monophosphate (2′,3′-cNADP^+^), and the resulting nicotinamide adenine dinucleotide phosphate (NADP^+^) is reduced to NADPH by glucose-6-phosphate dehydrogenase and used to transform glucose-6-phosphate to 6-phosphoglucanolactone. The quantity of NADPH formed during the coupled enzymatic reaction is a direct measure of CPDase activity [38].

The assay mixture contained 100 mM MES, pH 6.0, 3 mM MgCl_2_, 5 mM glucose-6-phosphate, and 0.6 U glucose-6-phosphate dehydrogenase. 500 ng of the purified enzyme was mixed with varying concentrations of the substrate, 2′,3′-cNADP^+^ (0, 0.05, 0.1, 0.2, 0.5, and 1.0 mM), and NADPH production was measured spectrophotometrically at 340 nm, using the absorption coefficient 6.22 cm^−1^mM^−1^. The measurements were carried out in triplicate at +25 °C using 96-well flat-bottom transparent plates with the TECAN Infinite M200 fluorescence spectrophotometer and iControl software.

### Thermal stability assay

Fluorescence-based thermal shift assays were carried out in triplicate, essentially as described [39]. Measurements were performed with a CFX96 RealTime PCR system (BioRad), using a 96-well thin-wall PCR plate. 1–5 μg of protein and 1–2 X SYPRO Orange dye were included in a total volume of 25 μl per condition. The plate was sealed with optical-quality adhesive film (BioRad) and heated from +25 °C to +99 °C in 0.5 °C increments. A total of 80 different conditions, varying in pH, salt type and concentration, and the presence of ligands, were screened. The fluorescence of SYPRO Orange was measured using excitation at 490 nm and emission at 575 nm. The apparent thermal melting points (T_m_) were determined for each construct under all 80 conditions.

### Small-angle X-ray scattering

SAXS measurements were carried out on the EMBL beamline X33 at DESY, Hamburg (Germany), the I911-4 SAXS beamline of MAX-Lab in Lund (Sweden), and the P12 BioSAXS beamline at PETRA-III, DESY, Hamburg. The *Sc*Trl1 PNK/CPDase and *Bf*PNK/CPDase eluted from SEC both as a dimer and as a monomer. These fractions were collected separately, concentrated, and used for SAXS measurements; only the data from the monomeric fraction were analyzed in detail, as dimerization was likely an artifact of non-specific disulphide formation. Sample concentrations were 1–10 mg/ml. Monomeric BSA was measured first as a MW standard. Solvent scattering from the corresponding buffer was measured identically before and after each sample, and the average background scattering was subtracted. The data were analyzed with the ATSAS [40] suite, as described [41, 42]. The data were processed using PRIMUS [43]. Distance distributions were calculated using GNOM [44], and *ab initio* bead modeling was done with DAMMIF [45]. DAMAVER [46] was used for model averaging. GASBOR [47] was used for building chain-like *ab initio* models, and MONSA [48] was used to assemble a 2-phase model of *Sc*Trl1 PNK/CPDase, employing data from both *Sc*Trl1 PNK/CPDase and *Sc*Trl1 CPDase. The models were compared in PyMOL.

## Results and discussion

### Protein expression and purification

**Fig. 1:**
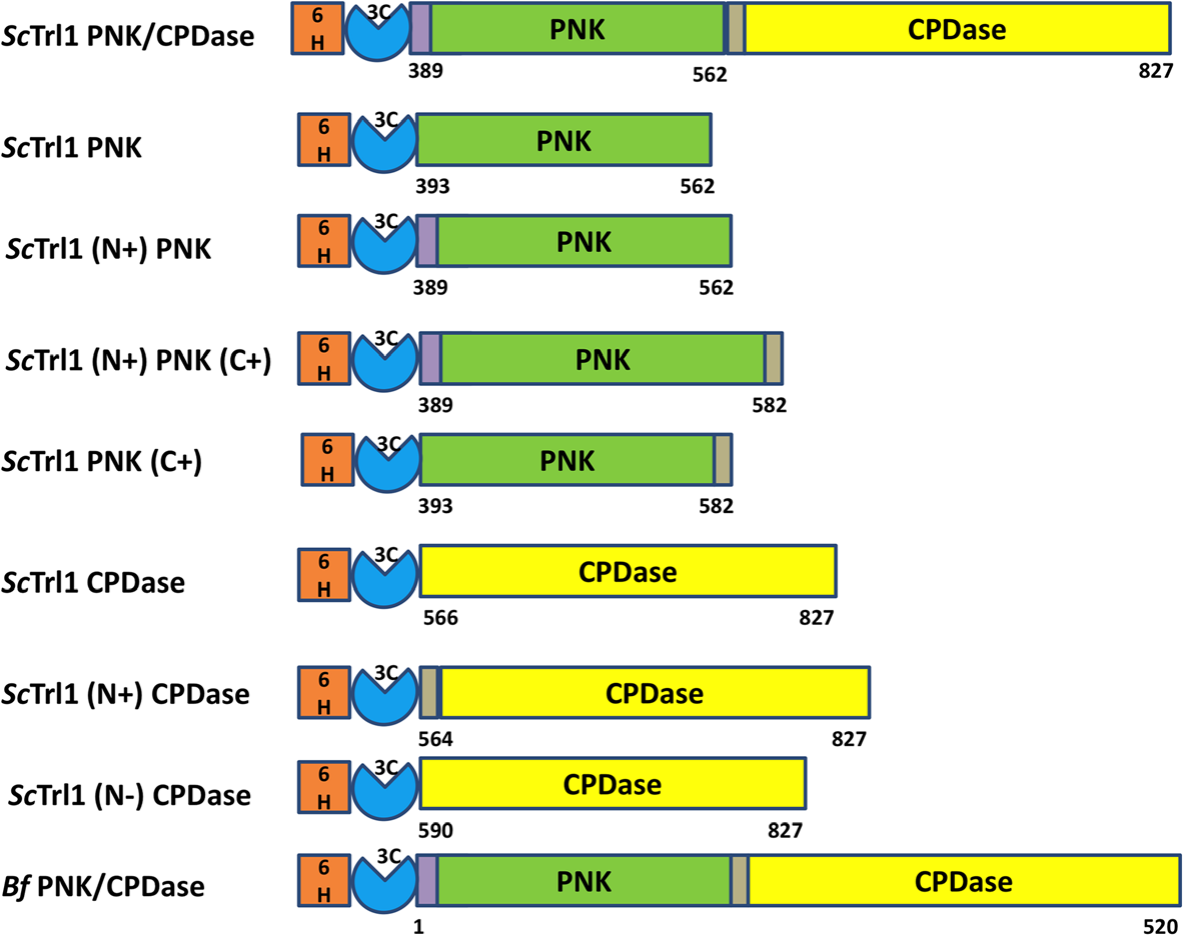
Preparation of expression constructs. Scheme of the constructs prepared for the kinase and phosphodiesterase domains of yeast Trl1 and for lancelet PNK/CPDase. 6H indicates the hexa-histidine tag, and 3C indicates the 3C protease cleavage site

A total of nine expression clones including eight for different segments of yeast Trl1 and one for lancelet PNK/CPDase were prepared (Fig. 1). All constructs contain an N-terminal hexahistidine tag followed by a 3C protease cleavage site and the corresponding PNK/CPDase domain. The expression level of the different constructs was screened in three *E. coli* cell lines [BL21(DE3), BL21(DE3) CodonPlus RIPL, and Rosetta(DE3)] under different expression conditions, including various inducer concentrations and post-induction growth temperatures. The constructs encoding different regions of the *Sc* PNK domain did not show any expression in the three cell lines tested; the remaining five constructs could be expressed. The same pattern was observed previously for the yeast *Sc*Trl1 PNK domain and the N-terminal domain of mouse CNPase [16, 30]. The solubility of the proteins expressed in Rosetta (DE3) was remarkably high. Screening growth conditions post-induction revealed longer incubation at lower temperatures to be the optimal expression strategy. For large-scale production of *Sc*Trl1 PNK/CPDase and *Bf*PNK/CPDase, up to 8 l of culture were used to obtain ~5 mg of protein, whereas 2 l of culture was sufficient to achieve similar quantities of the *Sc*CPDase domains.

**Fig. 2:**
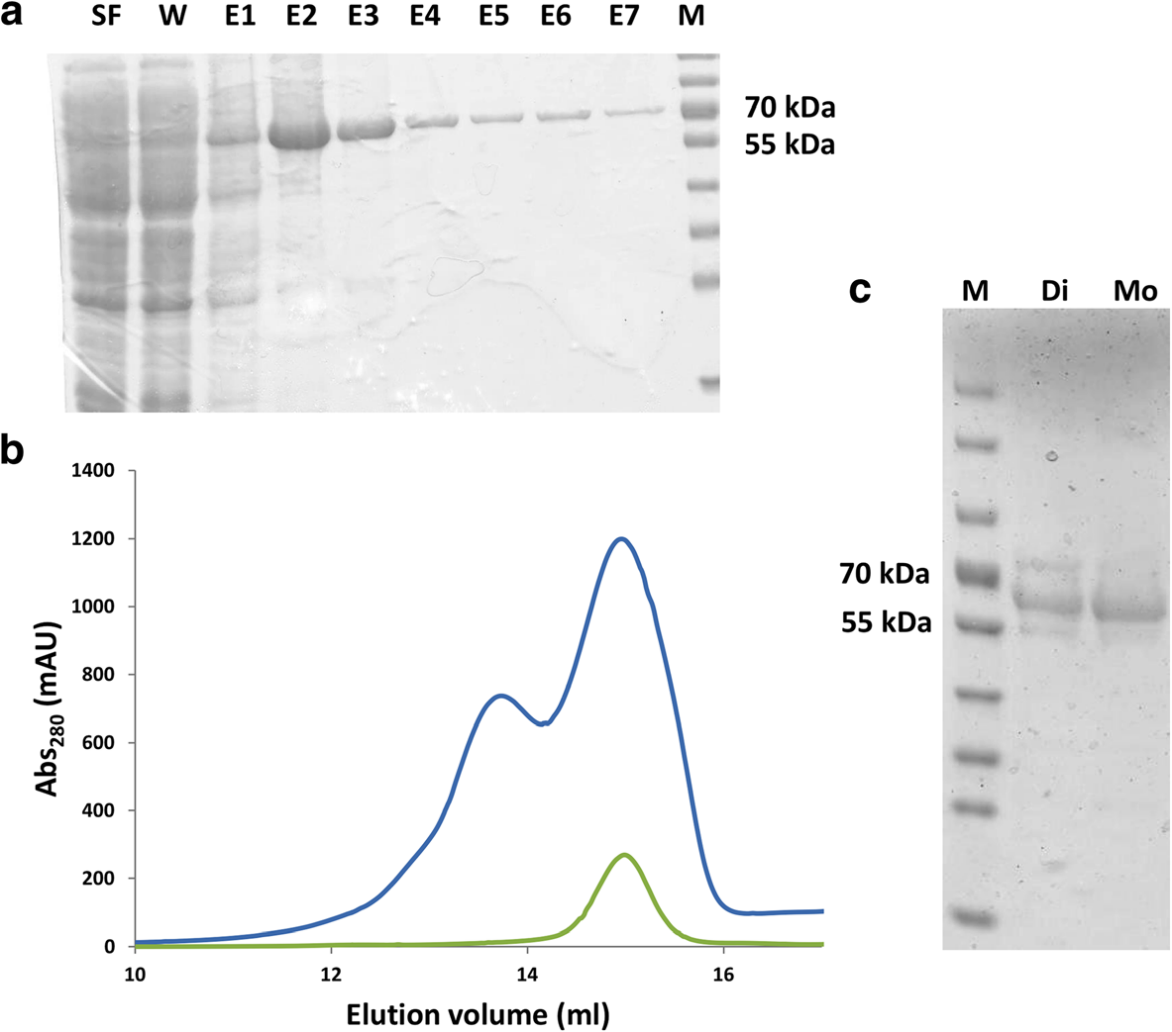
Purification of *Bf*PNK/CPDase. **a** SDS PAGE from a Ni-NTA purification of *Bf*PNK/CPDase. SF: supernatant flow-through, W: wash, E1-E7: fractions eluted with 500 mM imidazole, M: marker. The calculated size of the protein was 60.3 kDa. **b** Superdex75 SEC profile of *Bf*PNK/CPDase. The *blue curve* shows a mixture of monomer and dimer in the absence of any reducing agent, and the *green curve* shows the monomer in the presence of 5 mM β-ME. The single monomeric peak (*green*) indicates that the dimer formation is inhibited by β-ME. **c** SDS-PAGE from a SEC purification of *Bf*PNK/CPDase in the absence of a reducing agent. M: marker, Di: *Bf*PNK/CPDase dimer, Mo: *Bf*PNK/CPDase monomer

**Fig. 3:**
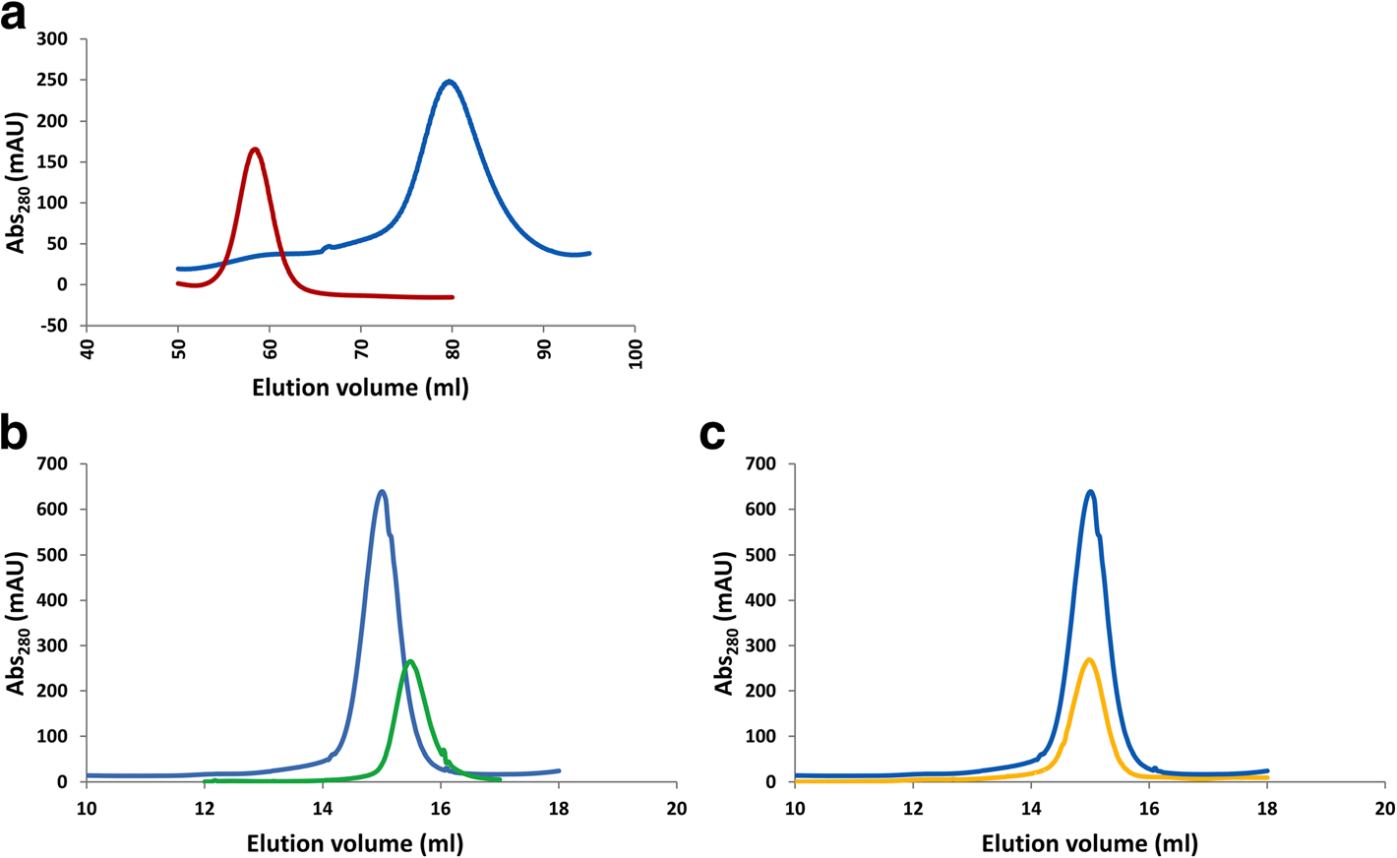
Yeast PNK/CPDase and CPDase purified by size exclusion chromatography. **a** Superdex200 SEC profile of *Sc*Trl1 PNK/CPDase monomer (*dark red*) and *Sc*Trl1 CPDase (*blue*). **b** Superdex75 SEC profile of *Sc*Trl1 CPDase (*blue*) and *Sc*Trl1 (N-) CPDase (*green*). **c** Superdex75 SEC profile of *Sc*Trl1 CPDase (*blue*) and *Sc*Trl1 (N+) CPDase (*orange*). The CPDase proteins elute around the same elution volume and exist as monomers

The expressed proteins were initially purified by Ni-NTA affinity chromatography and SEC (Additional file 2 & Figs. 2 and 3). *Sc*Trl1 PNK/CPDase and *Bf*PNK/CPDase eluted as double peaks with elution volumes corresponding to dimeric and monomeric forms. The fractions from each of the two peaks were collected separately, concentrated, and analyzed by SDS PAGE. Both peaks contained pure protein. The vertebrate homologue, mouse CNPase, exhibits similar oligomeric behaviour, and in this case, the N-terminal RNA-binding domain mediated dimerization [27]. The addition of either 10 mM β-ME or 5 mM DTT in the lysis and purification buffers inhibited dimerization of *Sc*Trl1 PNK/CPDase and *Bf*PNK/CPDase, indicating that the dimers are linked by a disulphide. Most likely the physiologically relevant form of each protein is monomeric.

The elution profiles of the CPDase constructs contained a single symmetric peak (Fig. 3b, c), and no oligomerization or RNA binding was detected. The absence of dimerization and RNA binding in the CPDase domain suggest that these processes require the N-terminal PNK domain. Similarly, the N-terminal PNK-like domain of mouse CNPase is involved not only in dimerization, but also in RNA binding [27]. The purified proteins were easily degraded upon storage at +4 °C. Hence, immediate flash-freezing and storage of the purified proteins in the freezer was essential. Since the stability of *Sc*Trl1 CPDase was found to be comparatively better than the other two CPDase protein variants, it was used in further biophysical and biochemical experiments.

### PNK/CPDase enzymes interact with *Eschericia coli* nucleic acids

**Fig. 4:**
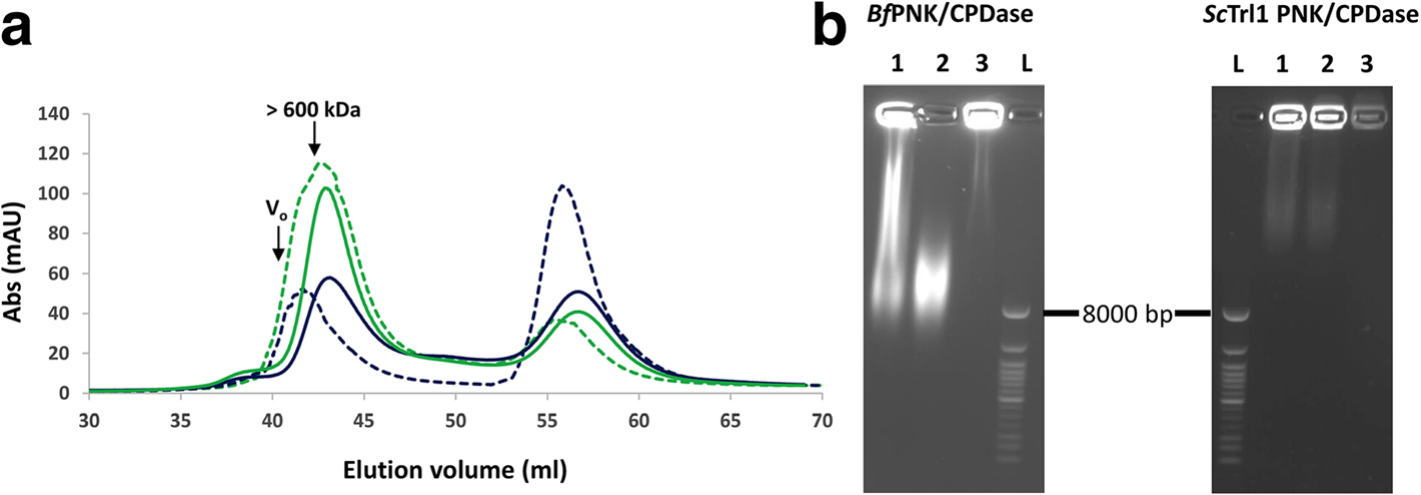
PNK/CPDase interacts with *E. coli* RNA. **a** Superdex 200 SEC profile of *Sc*Trl1 PNK/CPDase (*continuous lines*) and *Bf*PNK/CPDase (*dashed lines*). The peaks close to the void volume with A_260_ (*green*) higher than A_280_ (*blue*) indicate the presence of a nucleic acid. V_O_: column void volume. **b** Agarose gels of *Bf*PNK/CPDase and *Sc*Trl1 PNK/CPDase. L: DNA ladder, 1: The protein-nucleic acid complex eluted near the void volume from SEC, 2: DNase-treated protein-nucleic acid complex, 3: RNase-treated protein-nucleic acid complex. RNase degrades the nucleic acid, confirming that the co-purified molecule is, in fact, RNA

During the purification of *Sc*Trl1 PNK/CPDase and *Bf*PNK/CPDase, a large peak was always present right after the void volume of the SEC column (Fig. 4a). Since the absorbance at 260 nm was very high for this peak, co-purification of a nucleic acid, possibly RNA, from the expression host, was suspected. To identify the nucleic acid bound to the protein, the fractions eluted around the void volume in SEC were treated with DNase and RNase A. Agarose gels of nuclease-treated samples show that the bright smear in the larger elution fraction and in the same fraction treated with DNase was not found in the presence of RNase A (Fig. 4b). This finding confirms that the co-purified nucleic acid impurity contained RNA. In addition, DNA was present in the *Bf*PNK/CPDase sample. When a combination of RNase A and RNase T1 was added in the lysis buffer during subsequent purification, the A_260_ peak height remarkably decreased, additionally proving that the contaminant was RNA.

The behaviour of PNK/CPDase proteins in these assays was highly similar to the uracil-DNA degrading factor and mouse CNPase [27, 49]. CNPase has also been shown to interact with RNA *in vitro* and co-purify with poly(A)^+^ RNA; the catalytic domain of CNPase has been shown to be sufficient for binding with single stranded RNA homopolymers [50, 51]. However, a pulldown assay with mouse CNPase and poly(A)-sepharose indicated that the N-terminal domain binds RNA more efficiently than the C-terminal domain [27]. The absence of the peak closely following the void volume during SEC purification of the yeast CPDase domain indicates the N-terminal PNK domain is required for RNA binding, at least in the case of *E. coli* RNA.

### Bacterially expressed PNK/CPDase and CPDase proteins are folded

**Fig. 5:**
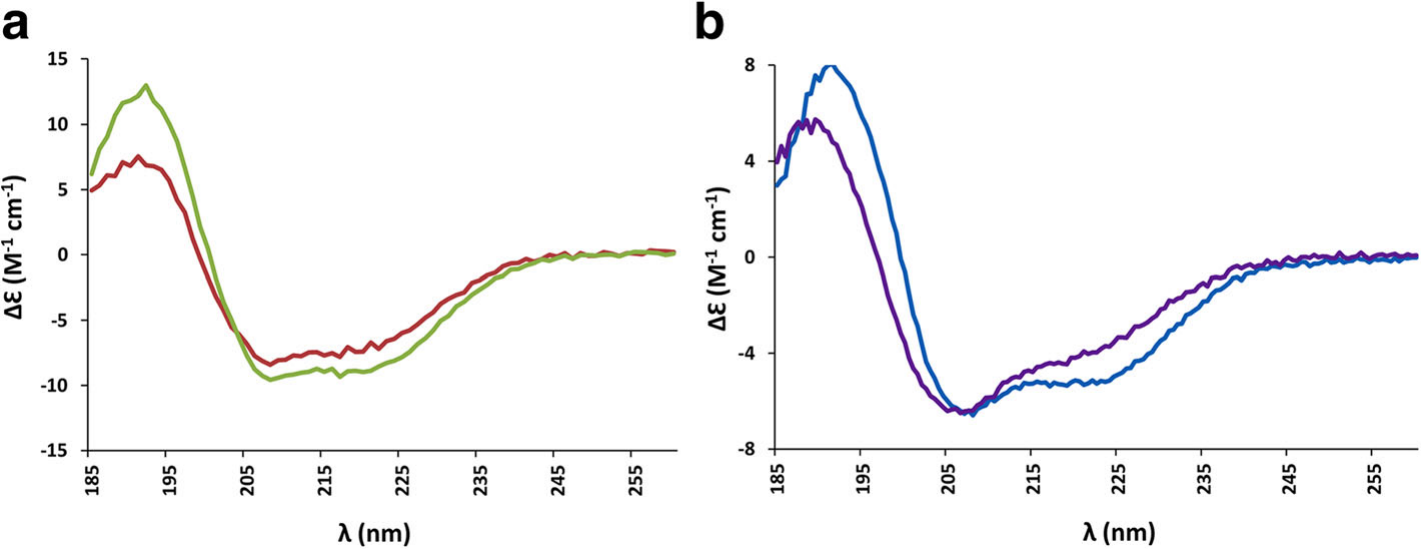
SRCD spectra of purified proteins. **a** *Sc*Trl1 PNK/CPDase (*red*) and *Bf*PNK/CPDase (*green*). **b** *Sc*Trl1 CPDase (*blue*) and *Sc*Trl1 (N-) CPDase (*purple*). The SRCD spectra indicate that all tested proteins are folded

The folding state of the purified proteins was analyzed by SRCD spectroscopy. Visual inspection of the SRCD spectra indicates that the PNK/CPDase and CPDase proteins were folded, containing a mixture of α helix and β strand. SRCD spectra from *Sc*Trl1 PNK/CPDase and *Bf*PNK/CPDase show a similar shape (Fig. 5a), indicating the presence of similar amounts of regular secondary structure. The spectra also indicate that the N-terminal truncation of *Sc*Trl1 CPDase is less folded than *Sc*Trl1 CPDase (Fig. 5b). The latter is indicative of sub-optimal folding of the truncated construct.

### Ligand binding improves thermal stability

Since the stability of the monomers of both *Sc*Trl1 PNK/CPDase and *Bf*PNK/CPDase during and after purification was initially low, the identification of a stabilizing buffer condition was necessary. A thermal stability assay was used to identify suitable buffer conditions that would offer increased stability of the protein. The melting curves and corresponding T_m_ values of *Sc*Trl1 PNK/CPDase, *Sc*Trl1 CPDase, and *Bf*PNK/CPDase indicate that all three proteins are most stable around pH 7.5. The combination of 50 mM HEPES, pH 7.5, and 150 mM NaCl was identified as an optimal stabilizing buffer.

**Fig. 6:**
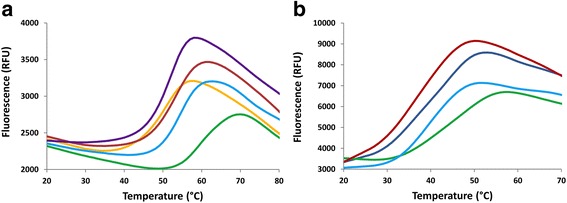
Thermal stability assay. **a** A selection of thermal shift assay melting curves for *Sc*Trl1 PNK/CPDase. Colour code: *Violet* - 50 mM citric acid, pH 5.5, 150 mM NaCl; *Dark red* - 50 mM MOPS, pH 6.5, 500 mM NaCl; *Orange* - 50 mM imidazole, pH 8.0, 150 mM NaCl; *Light blue* - 50 mM HEPES, pH 7.5, 150 mM NaCl; *Green* - 50 mM HEPES, pH 7.5, 150 mM NaCl, 1 mM 2′,3′-cNADP^+^. **b** A selection of thermal shift assay melting curves for *Bf* PNK/CPDase. Colour code: *Dark red* - 50 mM MOPS, pH 6.5, 500 mM NaCl; *Blue* - 50 mM MES, pH 7.0, 150 mM NaCl; *Light blue* - 50 mM HEPES, pH 7.5150 mM NaCl; *Green* - 50 mM HEPES, pH 7.5, 150 mM NaCl, 1 mM 2′,3′-cNADP^+^. A clear increase in the melting temperature can be observed, induced by buffer conditions with pH 7.5, low salt concentration, and 2′,3′-cNADP^+^

To analyze the effect of ligands on the thermal stability of the proteins, two known substrates of CPDase, 2′,3′-cCMP and 2′,3′-cNADP^+^, and the product NADP^+^ were included in the screen. The T_m_ values indicate that 2′,3′-cNADP^+^ improves the thermal stability of all three proteins tested (Fig. 6, Table 1); however, 2′,3′-cCMP and NADP^+^ do not influence the stability of any of them. The highest melting points obtained for *Sc*Trl1 PNK/CPDase and *Bf*PNK/CPDase were +63 °C and +48 °C, respectively, both in the presence of 2′,3′-cNADP^+^. *Sc*Trl1 CPDase was found to be the most stable among the proteins tested, with a melting temperature of +72 °C in a buffer containing 50 mM HEPES, pH 7.5, 150 mM NaCl, and 1 mM 2′,3′-cNADP^+^. Mouse CNPase is more stable at pH 5.5 with higher salt than at neutral pH with lower salt, which stabilizes the yeast and lancelet proteins [30]. The thermal stability of *Sc*Trl1 CPDase is also higher than that of the mouse CNPase catalytic domain and its mutated variants [28, 29].

**Table 1:** Comparison of T_m_ values for *Sc*Trl1 PNK/CPDase, *Sc*Trl1 CPDase, and *Bf*PNK/CPDase. The highest T_m_ values are indicated in bold

Protein	Buffer condition	T_m_ (°C)
*Sc*Trl1 PNK/CPDase	50 mM HEPES, pH 7.5, 150 mM NaCl	55
	50 mM imidazole, pH 8.0, 150 mM NaCl	50
	50 mM HEPES, pH 7.5, 150 mM NaCl, 1 mM 2′,3′-cNADP^+^	**63**
*Sc*Trl1 CPDase	50 mM HEPES, pH 7.5, 150 mM NaCl	58
	50 mM imidazole, pH 8.0, 150 mM NaCl	55
	50 mM HEPES, pH 7.5, 150 mM NaCl, 1 mM 2′,3′-cNADP^+^	**72**
*Bf*PNK/CPDase	50 mM HEPES, pH 7.5, 150 mM NaCl	43
	50 mM MES, pH 7.0, 500 150 mM NaCl	43
	50 mM HEPES, pH 7.5, 150 mM NaCl, 1 mM 2′,3′-cNADP^+^	**48**

### PNK/CPDase enzymes possess polynucleotide kinase activity

**Fig. 7:**
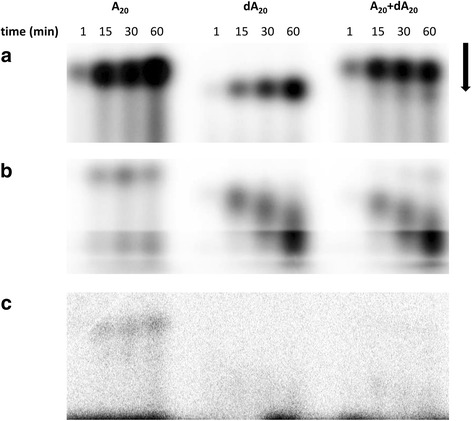
Polynucleotide kinase activity assay. PNK reaction mixtures of **a** *Sc*Trl1 PNK/CPDase, **b** *Bf*PNK/CPDase, and **c** *Sc*Trl1 CPDase. The reactions were carried out in the presence of Mg^2+^, with ATP as phosphate donor and with either A_20_, dA_20_, or both as acceptors. Both *Sc*PNK/CPDase and *Bf*PNK/CPDase display PNK activity with a clear substrate preference. The *Sc*CPDase domain alone is expectedly inactive. The *arrow* represents the direction of electrophoresis

The assay to test the PNK activity of the purified *Sc*Trl1 PNK/CPDase and *Bf*PNK/CPDase involves transfer of the ^32^P-labelled terminal phosphate from [γ^32^P] ATP to a 20-mer 5′-OH synthetic RNA (A_20_) or DNA oligonucleotide (dA_20_). To study the substrate preferences of the enzymes on RNA and DNA oligonucleotide substrates, the transfer of the radiolabelled phosphate to a mixture of A_20_ and dA_20_ was also analyzed. The time-dependent transfer of the phosphate from [γ^32^P] ATP to the 5′-OH groups of the synthetic oligonucleotides indicates that both *Sc*Trl1 PNK/CPDase and *Bf*PNK/CPDase possess PNK activity (Fig. 7a-b). *Sc*Trl1 CPDase did not catalyze phosphoryl transfer (Fig. 7c). The results indicate that *Sc*Trl1 PNK/CPDase prefers A_20_ (Fig. 7a) and *Bf*PNK/CPDase strongly prefers dA_20_ (Fig. 7b).

*Bf*PNK/CPDase was previously shown to prefer DNA over RNA, whereas another lancelet PNK, *Bf* Clp1, was reported to act exclusively on RNA [8]. This finding suggested a possible role for *Bf*PNK/CPDase in DNA repair. The fact that we saw DNA co-purifying with *Bf*PNK/CPDase and that the enzyme preferred the DNA substrate in the PNK assay support this hypothesis. The decrease in size of the labelled dA_20_ oligonucleotide during the assay could be related to an unknown activity of *Bf*PNK/CPDase towards DNA. The homologue of yeast and lancelet PNK/CPDase, mammalian CNPase, was found to rescue a yeast strain with an inactivating mutation in the CPDase domain of yeast tRNA ligase, but not a strain with a mutated kinase domain [52]. Thus, it appears that the N-terminal PNK-like domain of vertebrate CNPase is not functionally a PNK. This is supported by our results from the PNK activity assays. *Mm*CNPase, as well as its two domains separately, were inactive, while the homologous yeast and lancelet PNK/CPDase proteins were active (Fig. 7a, b, Additional file 3). This finding also supports the hypothesis that vertebrates might employ the 5′-P RNA ligation pathway only as an alternate to 3′-P RNA ligation and, thus, require only the exclusive polynucleotide kinase (Clp1) for the healing reaction, and not the N-terminal PNK-like domain of CNPase.

### PNK/CPDase and CPDase enzymes possess cyclic nucleotide phosphodiesterase activity

The CPDase activity of *Sc*Trl1 PNK/CPDase, *Bf*PNK/CPDase, and *Sc*Trl1 CPDase was assayed using 2′,3′-cNADP^+^ as substrate in a coupled enzyme assay [37, 38]. The results indicate that all tested constructs are active in the phosphodiesterase reaction (Table 2) and that the activity is comparable to that of the different constructs of mouse and rat CNPase. Thus, CPDase activity is a common denominator for 2H enzymes across kingdoms of life.

**Table 2:** Kinetic parameters for the CPDase activity of different constructs on 2′,3′-cNADP^+^

Protein	K_m_ (μM)	k_cat_ (s^−1^)	k_cat_/K_m_ (μM^−1^ s^−1^)
Enzymes used in the study			
*Bf*PNK/CPDase (500 ng)	440 ± 50	510 ± 20	1.15
*Sc*Trl1 PNK/CPDase (500 ng)	420 ± 35	317 ± 15	0.75
*Sc*Trl1 CPDase (500 ng)	237 ± 27	275 ± 18	1.16
Homologous enzymes			
*Mm*CNPase - full length (500 ng) [30]	193 ± 26	270 ± 10	1.40
*Mm*CNPase - catalytic domain (500 ng) [30]	445 ± 41	570 ± 19	1.28
Rat CNPase - full length [37]	263 ± 12	836 ± 11	3.2
Rat CNPase - catalytic fragment [37]	295 ± 22	1690 ± 39	5.7

### PNK/CPDase enzymes are elongated in solution

**Fig. 8:**
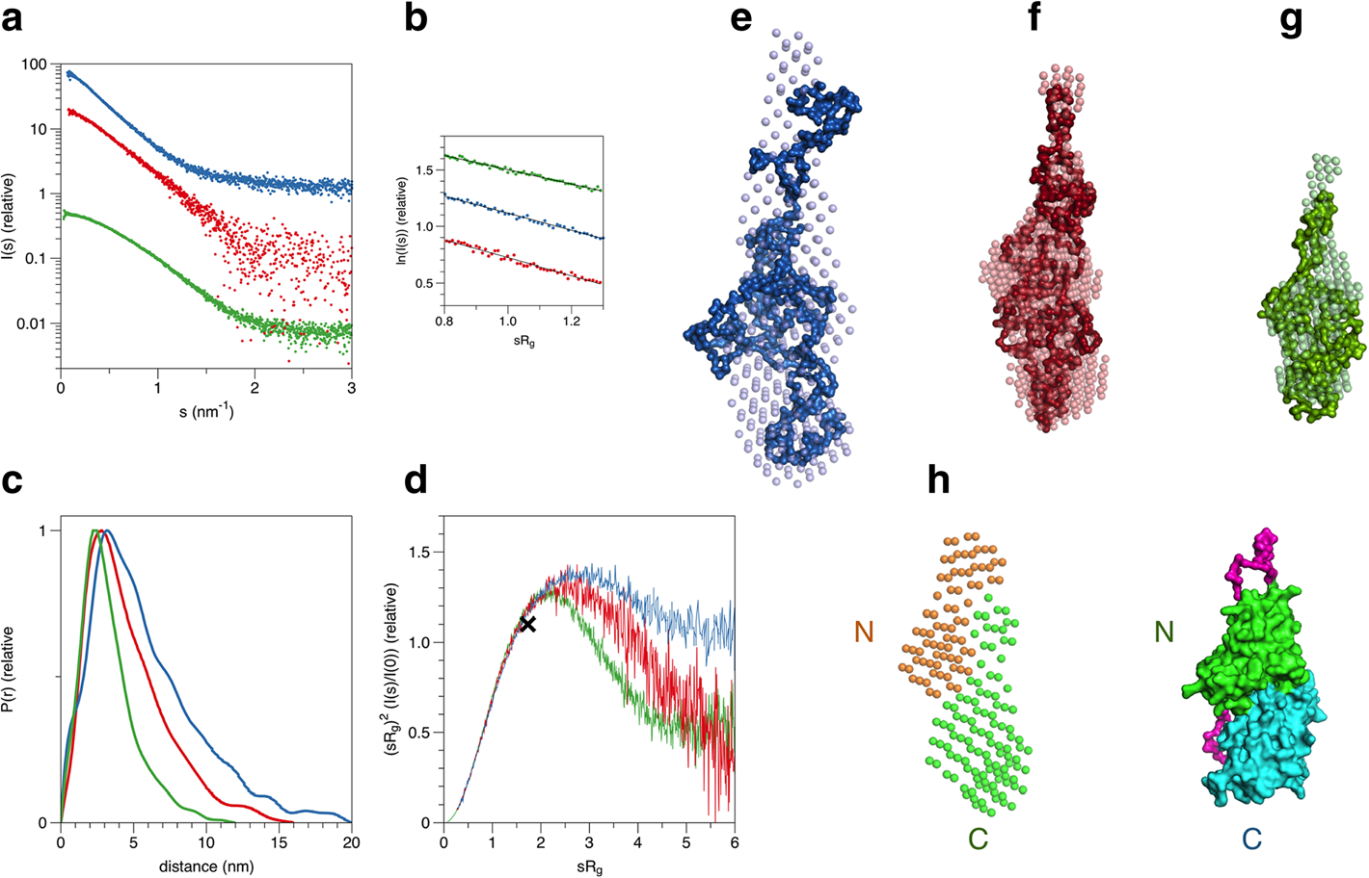
SAXS analysis of *Bf* and *Sc*Trl1 PNK/CPDases. **a** Scattering curves from monomeric *Bf* (*blue*) and *Sc*Trl1 (*red*) PNK/CPDases and the *Sc*Trl1 CPDase domain (*green*). The curves have been displaced for clarity. **b** Guinier plots for the samples in (A), plotted between 0.8 < sR_g_ < 1.3. The curves have been moved in the y dimension for clarity. **c** Distance distribution functions for the samples shown in (A). **d** Dimensionless Kratky plots. The *cross marks* the expected peak position for a folded globular protein (x = 1.732, y = 1.1). *Bf*PNK/CPDase is most flexible of the proteins. **e-g** *Ab initio* 3D models for *Bf*PNK/CPDase, *Sc*Trl1 PNK/CPDase, and the *Sc*Trl1 CPDase domain, respectively. The DAMMIF bead model is shown by spheres and the GASBOR chain-like model as a surface in each panel. **h** Comparison of the 2-phase MONSA model of *Sc*Trl1 PNK/CPDase (*left*) with the earlier published SAXS structure of full-length *Mm*CNPase (*right*) [28]. The positions of the N-terminal PNK domain and the C-terminal CPDase domain are indicated for both proteins. See Table 3 for the chi^2^ values corresponding to the fit between the raw data and the models

SAXS measurements were carried out to determine low-resolution structural models of the proteins and to analyze their oligomeric state in solution. Although SAXS data were collected for all the five expressed constructs, only three of them, the *Sc*Trl1 PNK/CPDase, the *Sc*Trl1 CPDase, and the *Bf*PNK/CPDase, yielded good-quality SAXS data, and only these were used for further processing and modeling. The best datasets were collected between 3 and 4 mg/ml, with a strong scattering signal and without significant interparticle effects, as shown by the linearity of the Guinier plot (Fig. 8a, b).

The monomeric *Sc*Trl1 PNK/CPDase and *Bf*PNK/CPDase display similarly elongated conformations, with *Bf*PNK/CPDase being more extended (Fig. 8a, e, f and Table 3). The estimated Porod volume is in agreement with a monomeric form of both proteins. The *p*(r) profile displays a single peak with shoulders for both yeast and lancelet PNK/CPDase proteins (Fig. 8c), indicating an asymmetric scattering particle, possibly containing two domains bridged by a flexible linker. Dimensionless Kratky plots (Fig. 8d) further show that *Bf*PNK/CPDase is the most flexible of the studied samples, in line with its relatively large volume-to-mass ratio (Table 3).

**Table 3:** Structural parameters derived from experimental SAXS data and comparison to known parameters of mouse CNPase

Protein	*Sc*Trl1 PNK/CPDase	*Bf*PNK/CPDase	*Sc*Trl1 CPDase	*Mm*CNPase [28]	*Mm*CNPase catalytic domain [27]
*D*_*max*_ (nm)	11.6	14.3	8.5	8.8	7.1
Guinier *R*_*g*_ (nm)	3.37	4.16	2.48	2.6	2.13
Real-space *R*_*g*_ (nm)	3.79	4.83	2.69	-	-
V_P_ (nm^3^)	78.6	137.7	50.1	-	44.4
*Ab initio* V (nm^3^)/estimated MW of globular particle (kDa)	106/78.7	212/157.4	30.5/22.6	-	-
Monomeric MW (kDa)	52.7	60.3	32.2	42	24
s_min_/s_max_ (nm^−1^) bead-based modeling	0.09-2.52	0.12-1.31	0.08-3.72	-	-
s_min_/s_max_ (nm^−1^) chain-based modeling	0.09-5.0	0.12-2.8	0.08-3.72	-	-
Chi^2^	1.0 (DAMMIF), 0.7 (GASBOR), 1.3 (MONSA)	1.0 (DAMMIF), 1.0 (GASBOR)	0.6 (DAMMIF), 0.7 (GASBOR), 1.2 (MONSA)	-	-

The 3D models for both PNK/CPDases are consistent with an arrangement, where the two domains form an elongated assembly. The model suggests that the active sites of the PNK domain and CPDase domain are accessible and not being blocked by the neighbouring domain. An open active site facilitates substrate binding without the need for large conformational changes. If the domains would cover each other more extensively, the shape of the molecule would be more globular.

**Fig. 9:**
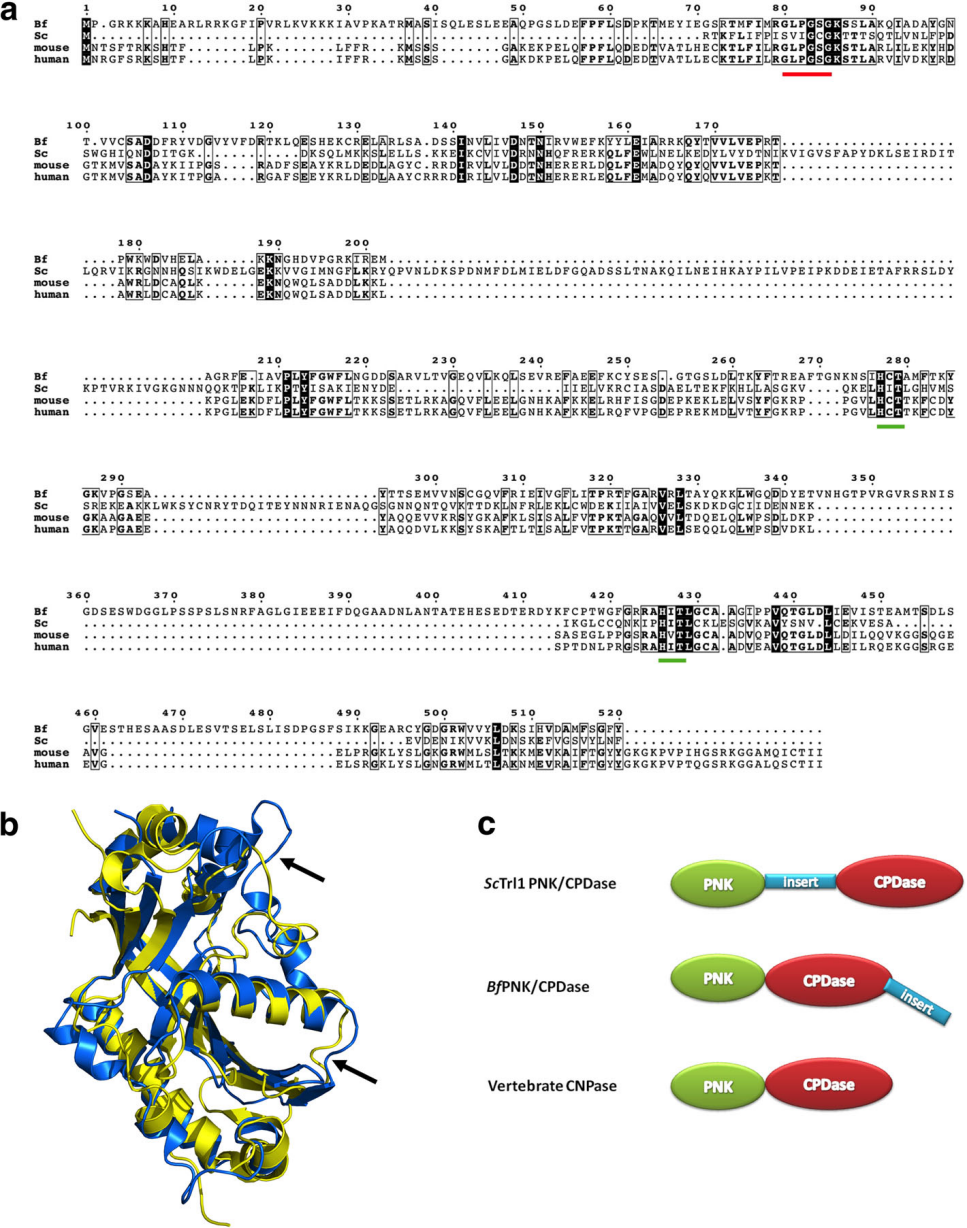
Domain arrangement of PNK/CPDase proteins. **a** Multiple sequence alignment using T-Coffee [54]. Aligned sequences of *Sc*Trl1 PNK/CPDase (*Sc*), *Bf*PNK/CPDase (*Bf*), *Mm*CNPase (mouse), and *Hs*CNPase (human). The N-terminal P-loop motif and two C-terminal H-x-(T/S)-x motifs are underlined in *red* and *green*, respectively. **b** Superposition of the homology model of the CPDase domain of *Bf*PNK/CPDase (*blue*), generated using *Phyre* ^2^, and the crystal structure of the catalytic domain of *Mm*CNPase (*yellow*) [PDB ID: 2YDB] [27, 53]. The *black arrows* point to the locations of flexible loops in the homology model of the CPDase domain of *Bf*PNK/CPDase (*blue*). **c** The overall three-dimensional shapes of PNK/CPDase proteins determined in this study can be used to propose a structural arrangement of the domains

The solution structure of full-length mouse CNPase is similar to that of the monomeric *Sc*Trl1 PNK/CPDase and *Bf*PNK/CPDase [28], although somewhat more compact, reflecting the presence of large flexible insertions in the yeast and lancelet enzymes. Mouse CNPase is a monomer with an elongated conformation in solution [28]. Although the molecular masses of monomeric *Sc*Trl1 PNK/CPDase [50.52 kDa] and monomeric mouse CNPase [44.8 kDa] are close to each other, the *D*_*max*_ and *R*_*g*_ values are comparatively higher for the *Sc*Trl1 PNK/CPDase (Table 3). This could be due to the presence of a flexible linker region between the N-terminal PNK and C-terminal CPDase domains in *Sc*Trl1 PNK/CPDase: this segment is absent in mouse CNPase (Fig. 9a). Based on SAXS data and molecular modelling, the C-terminal membrane-anchoring tail of the mouse CNPase lies in the middle region of the molecule, enabling the association of the active site to the vicinity of the lipid bilayer [28].

### CPDase is compact in solution

The molecular size determined from the SAXS profile of *Sc*Trl1 CPDase indicates that the molecule is more compact in solution than *Sc*Trl1 PNK/CPDase and exists as a monomer (Fig. 8a, c, d, f & g, Table 3). Molecular mass determination shows the presence of a monomer. The Porod volume is also in line with the presence of monomeric *Sc*Trl1 CPDase. The SAXS model shows the presence of an extension presumably in the N terminus of the protein; this tail possibly arises from the interdomain linker at the N terminus of the construct, suggesting the requirement for a shorter construct of the protein without the N terminal insertion to facilitate crystallization.

The solution structure of *Sc*Trl1 CPDase resembles that of the catalytic domain of mouse CNPase [27]. The mouse CNPase catalytic domain is also monomeric in solution. Both *Sc*Trl1 CPDase and the catalytic domain of mouse CNPase adopt an elongated conformation possibly due to the opening of the active-site cleft, as found in the NMR structure of rat CNPase catalytic domain [25, 27]. The mouse CNPase catalytic domain displays a more compact structure in the presence of citrate and the ligand also reduces aggregation of the protein [27]. It has been suggested that the overall solution conformation of fully active CNPase might be more open than that seen in the crystal structures, in which the enzyme is bound to citrate and sulphate [27]. Different lengths of the mouse CNPase catalytic domain have been studied by SAXS [27]. The presence of a C-terminal tail does not alter the conformation of the catalytic domain in solution, whereas N-terminally extended CNPase catalytic domain has a higher radius of gyration. Both N- and C-terminally extended protein is remarkably more elongated [27]. The symmetric peak of the *p*(r) for *Sc*Trl1 CPDase shows that the protein, similarly to the mouse CNPase catalytic domain, is more compact than the protein variants that also contain the N-terminal domain (Fig. 8c).

The solution shapes and behaviour of the PNK/CPDases and the CPDase are in many aspects comparable to those of the full-length mouse CNPase and its catalytic domain, respectively. The differences are likely caused by the relatively large insertions compared to CNPase: the yeast enzyme has a long insertion between the PNK and CPDase domains, while the lancelet enzyme has insertions in two loop regions of the CPDase domain (Fig. 9a, b). The SAXS model of full-length mouse CNPase indicates that the N-terminal PNK-like and the C-terminal phosphodiesterase domains form an elongated assembly [28], and as shown here, the same is true for the yeast and lancelet enzymes. The biphasic model built for the yeast enzyme (Fig. 8h) compares well to the mouse CNPase solution structure. Thus, the domain arrangements of the yeast and lancelet PNK/CPDase in solution resemble that of CNPase. An open elongated conformation of the full-length protein could be crucial for substrate binding [28]. Superposition of a homology model of the CPDase domain of *Bf*PNK/CPDase and the catalytic domain of *Mm*CNPase shows that the active site consisting of the 2*H* motifs is structurally conserved [27, 53] (Fig. 9b). The homology model of the lancelet CPDase domain contains a symmetrical bilobed tertiary structure, which is similar to the crystal structures of the catalytic domain of mouse and human CNPase [26–29]. The inserted loops within the CPDase domain of *Bf*PNK/CPDase (Fig. 9b) were also the most flexible in the recently-determined structure of a bacterial 2*H* phosphoesterase, LigT from *E. coli* [51].

Comparison of the SAXS models of *Sc*Trl1 PNK/CPDase, *Bf*PNK/CPDase, and *Sc*Trl1 CPDase with the SAXS structural parameters of the homologous mouse CNPase indicates that the structural and hydrodynamic properties of the different constructs can be explained by the presence of sequence insertions in the yeast and lancelet enzymes. A structural model of the PNK/CPDase proteins (Fig. 9c) can be proposed based on the determined 3-dimensional shapes. Yeast PNK/CPDase exhibits an elongated conformation with the presence of a possibly flexible region between the N-terminal PNK and C-terminal CPDase domains. Removal of the flexible region, which is not present in mouse CNPase, might aid in crystallization of the domains separately. The model also indicates that the two loop insertions in the CPDase domain in lancelet PNK/CPDase result in an even more elongated molecule, and these long loops point away from the PNK domain at the other end of the molecule. It is possible that *Bf*PNK/CPDase also has a flexible region at its N terminus.

## Conclusions

The open elongated conformation of the PNK/CPDases might play a role in RNA substrate binding; however, this needs to be confirmed by high-resolution structures of the enzymes with bound substrates. Based on currently available structural data, we can conclude that both enzymes display structural similarities to other members of the 2*H*-phosphoesterase family - not only at the level of conserved sequence motifs, but also in their respective domain arrangements. The yeast and lancelet healing enzymes possess both PNK and CPDase activities, whereas the mouse CNPase, which also binds RNA, has no PNK activity. This suggests a loss of function at the CNPase N-terminal PNK-like domain over the course of evolution. On the other hand, *Bf*PNK/CPDase may have a unique substrate specificity within the family with respect to its PNK activity. Thus, our results on the structure and function of tRNA healing enzymes from yeast and lancelet provide evidence supporting the evolution of vertebrate CNPase from functional tRNA healing enzymes.

## Additional files

Additional file 1: Table S1. Primers used for PCR amplification of target DNA fragments. (PDF 54 kb)

Additional file 2: Figure S1. The yeast PNK/CPDase and CPDase proteins purified by Ni-NTA affinity chromatography. A) SDS-PAGE from a Ni-NTA purification of *Sc*Trl1 PNK/CPDase. M: marker, E1-E8: fractions eluted with 500 mM imidazole. The calculated size of the protein was 52.7 kDa. B) SDS-PAGE from Ni-NTA purifications of *Sc*Trl1 CPDase and *Sc*Trl1 (N+) CPDase. M: marker, SF1 and W1 are supernatant and wash flow-through samples from the purification of *Sc*Trl1 CPDase, E1-E5: *Sc*Trl1 CPDase fractions eluted with 500 mM imidazole. The calculated size of the protein was 32.2 kDa. SF2 and W2 are supernatant and wash flow-through samples from the purification of *Sc*Trl1 (N+) CPDase, E6-E10: *Sc*Trl1 (N+) CPDase fractions eluted with 500 mM imidazole. The calculated size of the protein was 32.4 kDa. C) SDS PAGE from a Ni-NTA purification of *Sc*Trl1 (N-) CPDase. SF: supernatant flow-through, W: wash flow-through, E1-E10: fractions eluted with 500 mM imidazole, M: marker. The calculated size of the protein was 29.6 kDa. (PDF 220 kb)

Additional file 3: Figure S2. Polynucleotide kinase activity assay. PNK reaction mixtures of A) T4 PNK, B) *Mm*CNPase C) *Mm*CNP_N and D) *Mm*CNP_C. The reactions were carried out in the presence of Mg^2+^, with ATP as phosphate donor and with either A_20_, dA_20_, or both. T4 PNK was used as a positive control to validate the assay setup. The *Mm*CNP domains were tested separately, with a negative result in the chosen assay conditions. The arrow represents the direction of electrophoresis. (PDF 387 kb)

## Abbreviations

*Bf*: *Branchiostoma floridae*
BSA: Bovine serum albumin
cCMP: cyclic cytosine monophosphate
CNPase: 2’,3’-cyclic nucleotide 3’-phosphodiesterase
CNS: Central nervous system
CPDase: Cyclic phosphodiesterase
DEPC: Diethylpyrocarbonate
DESY: Deutsches Elecktronen-Synchrotron
*D*_*max*_: maximum dimension of a particle
DNA: Deoxyribonucleic acid
DNase: Deoxyribonuclease
DTT: Dithiothreitol
*E. coli*: *Eschericia coli*
EDTA: Ethylenediaminetetraacetic acid
EMBL: European Molecular Biology Laboratory
GTP: Guanosine triphosphate
HEPES: Hydroxyethyl piperazineethanesulfonic acid
HIV: Human immunodeficiency virus
*Hs*: *Homo sapiens*
IPTG: Isopropyl β-D-1-thiogalactopyranoside
*Mm*: *Mus musculus*
NADP^+^: Oxidized form of nicotinamide adenine dinucleotide phosphate
NADPH: Reduced form of nicotinamide adenine dinucleotide phosphate
PCR: Polymerase chain reaction
PDB: Protein Data Bank
PNK: Polynucleotide kinase
PNS: Peripheral nervous system
Poly-A: Polyadenylate
*R*_*g*_: Radius of gyration
RNA: Ribonucleic acid
RNase: Ribonuclease
SAXS: Small-angle X-ray scattering
*Sc* or *S. cerevisiae*: *Saccharomyces cerevisiae*
SDS-PAGE: Sodium dodecyl sulphate-polyacrylamide gel electrophoresis
SEC: Size exclusion chromatography
SLIC: Sequence and ligation independent cloning
SRCD: Synchrotron radiation circular dichroism
Trl1: tRNA ligase 1
tRNA: transfer ribonucleic acid
β-ME: Beta-mercaptoethanol.
