# Supplementary material for: Structural similarities and functional differences clarify evolutionary relationships between tRNA healing enzymes and the myelin enzyme CNPase
Source: BMC Biochem. 2017 May 16;18:7. doi: 10.1186/s12858-017-0084-2 (PMC5434554; doi:10.1186/s12858-017-0084-2)
Supplement: Supplementary file 1 — Primers used for PCR amplification of target DNA fragments. (PDF 54 kb) [file 12858_2017_84_MOESM1_ESM.pdf]

Supplementary Table 1. Primers used for PCR amplification of target DNA fragments

Construct	Primers designed
*Sc*Trl1 PNK/CPDase	**Fwd:** 5'-cagggacccggtCGCACTAAATTTTTAATCTT-3'<br>**Rev:** 5'-cgaggagaagcccggttaAAAATTTAAATATACACTGC-3'
*Sc*Trl1 PNK	**Fwd:** 5'-cagggacccggtTTAATCTTTCCAATATCAGT-3'<br>**Rev**: 5'-cgaggagaagcccggttaTCCAAAATCTAACTCTATCA-3'
*Sc*Trl1 (N+) PNK	**Fwd**: 5'-cagggacccggtCGCACTAAATTTTTAATCTT-3'<br>**Rev:** 5'-cgaggagaagcccggttaTCCAAAATCTAACTCTATCA-3'
*Sc*Trl1 (N+) PNK (C+)	**Fwd:** 5'-cagggacccggtCGCACTAAATTTTTAATCTT-3'<br>**Rev:** 5'-cgaggagaagcccggttaATAAGCTTTATGAATTTCAT-3'
*Sc*Trl1 PNK (C+)	**Fwd:** 5'-cagggacccggtTTAATCTTTCCAATATCAGT-3'<br>**Rev:** 5'-cgaggagaagcccggttaATAAGCTTTATGAATTTCAT-3'
*Sc*Trl1 CPDase	**Fwd:** 5'-cagggacccggtTCTTCATTAACCAATGCGAA-3'<br>**Rev:** 5'-cgaggagaagcccggttaAAAATTTAAATATACACTGC-3'
*Sc*Trl1 (N+) CPDase	**Fwd:** 5'-cagggacccggtGCAGACTCTTCATTAACCAA-3'<br>**Rev:** 5'-cgaggagaagcccggttaAAAATTTAAATATACACTGC-3'
*Sc*Trl1 (N-) CPDase	**Fwd:** 5'-cagggacccggtCCGAAAGATGATGAAATTGA-3'<br>**Rev:** 5'-cgaggagaagcccggttaAAAATTTAAATATACACTGC-3'
*Bf*PNK/CPDase	**Fwd:** 5'-cagggacccggtATGCCAGGAAGGAAGAAAAA-3'<br>**Rev:** 5'-cgaggagaagcccggttaATAGAAGCCTGAAAACATTG-3'
