# Supplementary material for: Structural similarities and functional differences clarify evolutionary relationships between tRNA healing enzymes and the myelin enzyme CNPase
Source: BMC Biochem. 2017 May 16;18:7. doi: 10.1186/s12858-017-0084-2 (PMC5434554; doi:10.1186/s12858-017-0084-2)
Supplement: Supplementary file 2 — The yeast PNK/CPDase and CPDase proteins purified by Ni-NTA affinity chromatography. A) SDS-PAGE from a Ni-NTA purification of ScTrl1 PNK/CPDase. M: marker, E1-E8: fractions eluted with 500 mM imidazole. The calculated size of the protein was 52.7 kDa. B) SDS-PAGE from Ni-NTA purifications of ScTrl1 CPDase and ScTrl1 (N+) CPDase. M: marker, SF1 and W1 are supernatant and wash flow-through samples from the purification of ScTrl1 CPDase, E1-E5: ScTrl1 CPDase fractions eluted with 500 mM imidazole. The calculated size of the protein was 32.2 kDa. SF2 and W2 are supernatant and wash flow-through samples from the purification of ScTrl1 (N+) CPDase, E6-E10: ScTrl1 (N+) CPDase fractions eluted with 500 mM imidazole. The calculated size of the protein was 32.4 kDa. C) SDS PAGE from a Ni-NTA purification of ScTrl1 (N-) CPDase. SF: supernatant flow-through, W: wash flow-through, E1-E10: fractions eluted with 500 mM imidazole, M: marker. The calculated size of the protein was 29.6 kDa. (PDF 220 kb) [file 12858_2017_84_MOESM2_ESM.pdf]

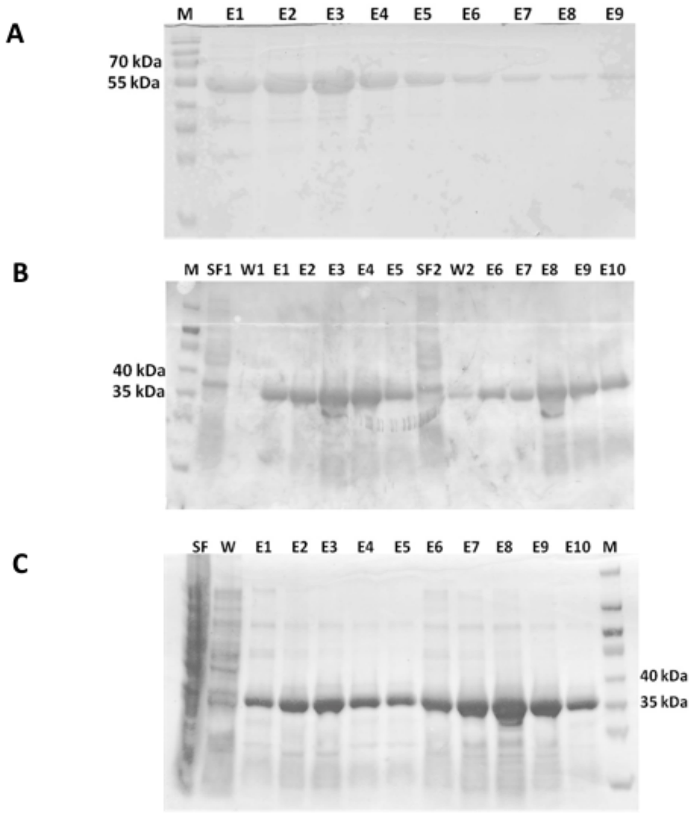

Supplementary Figure 1. The yeast PNK/CPDase and CPDase proteins purified by Ni-NTA affinity chromatography. A) SDS-PAGE from a Ni-NTA purification of *Sc*Trl1 PNK/CPDase. M: marker, E1-E8: fractions eluted with 500 mM imidazole. The calculated size of the protein was 52.7 kDa. **B)** SDS-PAGE from Ni-NTA purifications of *Sc*Trl1 CPDase and *Sc*Trl1 (N+) CPDase. M: marker, SF1 and W1 are supernatant and wash flow-through samples from the purification of *Sc*Trl1 CPDase, E1-E5: *Sc*Trl1 CPDase fractions eluted with 500 mM imidazole. The calculated size of the protein was 32.2 kDa. SF2 and W2 are supernatant and wash flow-through samples from the purification of *Sc*Trl1 (N+) CPDase, E6-E10: *Sc*Trl1 (N+) CPDase fractions eluted with 500 mM imidazole. The calculated size of the protein was 32.4 kDa. **C)** SDS PAGE from a Ni-NTA purification of *Sc*Trl1 (N-) CPDase. SF: supernatant flow-through, W: wash flow-through, E1-E10: fractions eluted with 500 mM imidazole, M: marker. The calculated size of the protein was 29.6 kDa.
