# Supplementary material for: Structural similarities and functional differences clarify evolutionary relationships between tRNA healing enzymes and the myelin enzyme CNPase
Source: BMC Biochem. 2017 May 16;18:7. doi: 10.1186/s12858-017-0084-2 (PMC5434554; doi:10.1186/s12858-017-0084-2)
Supplement: Supplementary file 3 — Polynucleotide kinase activity assay. PNK reaction mixtures of A) T4 PNK, B) MmCNPase C) MmCNP_N and D) MmCNP_C. The reactions were carried out in the presence of Mg2+, with ATP as phosphate donor and with either A20, dA20, or both. T4 PNK was used as a positive control to validate the assay setup. The MmCNP domains were tested separately, with a negative result in the chosen assay conditions. The arrow represents the direction of electrophoresis. (PDF 387 kb) [file 12858_2017_84_MOESM3_ESM.pdf]

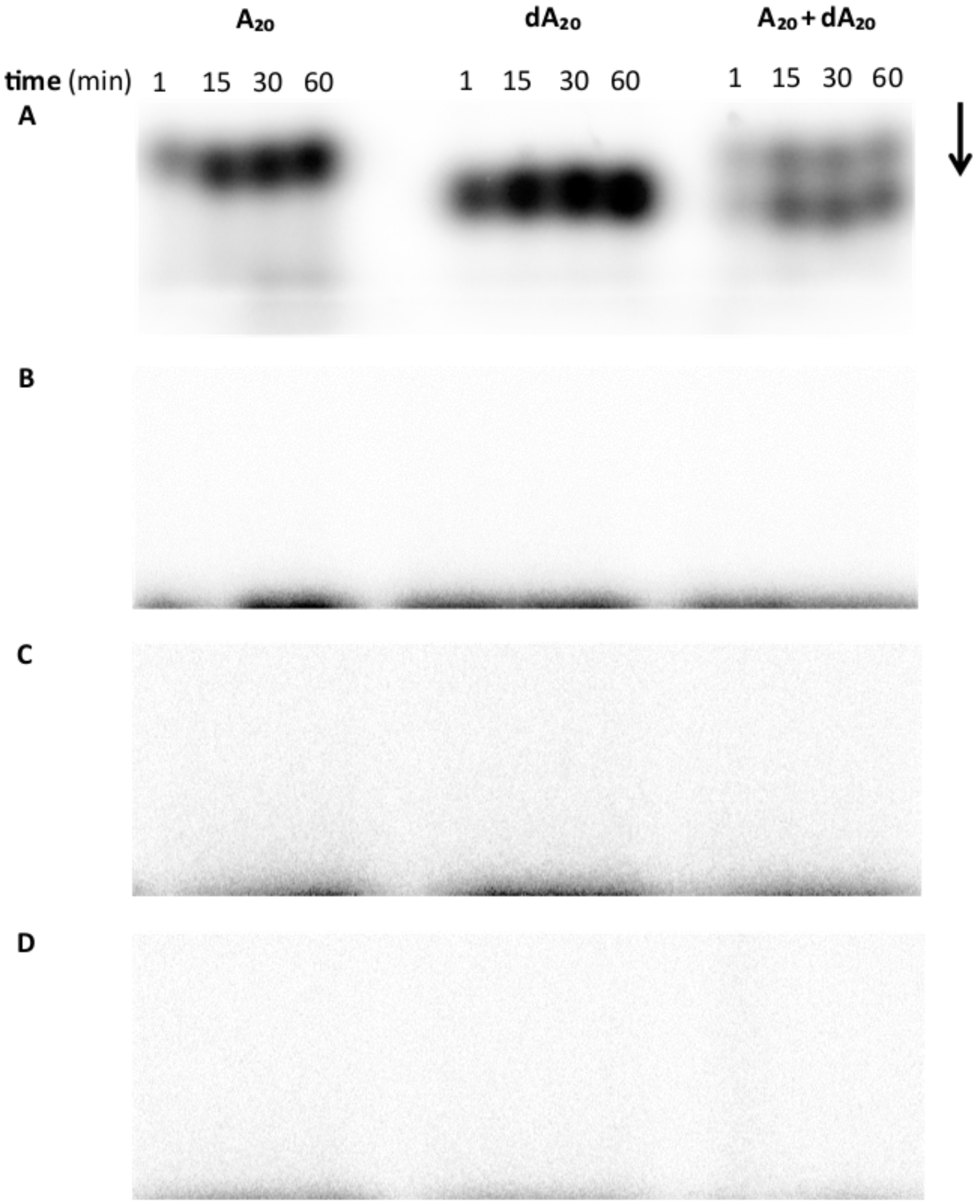

Supplementary Figure 2. Polynucleotide kinase activity assay. PNK reaction mixtures of **A)** T4 PNK, **B)** *Mm*CNPase, **C)** *Mm*CNP_N, and **D)** *Mm*CNP_C. The reactions were carried out in the presence of Mg^{2+}, with ATP as phosphate donor and with either A_{20}, dA_{20}, or both. T4 PNK was used as a positive control to validate the assay setup. The *Mm*CNP domains were tested separately, with a negative result in the chosen assay conditions. The arrow represents the direction of electrophoresis.
